# Investigating pathogenic SNP of PKCι in HCV-induced hepatocellular carcinoma

**DOI:** 10.1038/s41598-023-39804-0

**Published:** 2023-08-02

**Authors:** Naila Khan, Khushbukhat Khan, Yasmin Badshah, Janeen H. Trembley, Naeem Mahmood Ashraf, Maria Shabbir, Lubna Danish, Tayyaba Afsar, Ali Almajwal, Saira Justin, Zafarul Hasan, Suhail Razak

**Affiliations:** 1 Department of Healthcare Biotechnology, Atta-Ur-Rahman School of Applied Biosciences (ASAB), National University of Sciences and Technology (NUST), Islamabad, Pakistan; 2 Research Service, Minneapolis VA Health Care System, Minneapolis, MN USA; 3 Department of Laboratory Medicine and Pathology, University of Minnesota, Minneapolis, MN USA; 4 Masonic Cancer Center, University of Minnesota, Minneapolis, MN USA; 5 School of Biochemistry and Biotechnology, University of the Punjab, Lahore, Pakistan; 6 Agricultural Research Institute, Tarnab, Peshawar, Pakistan; 7 Department of Community Health Sciences, College of Applied Medical Sciences, King Saud University, Riyadh, Saudi Arabia; 8 College of Nursing, Sciences, King Saud University, Riyadh, Saudi Arabia

**Keywords:** Cancer, Computational biology and bioinformatics

## Abstract

Hepatocellular carcinoma is a leading cause of cancer-related deaths due to its complexity in diagnosis, chemo-resistance, and aggressive nature. Identifying pathogenic single nucleotide polymorphism (SNP) in protein kinase C iota (PKCι) can be a potential biomarker in the prognosis and treatment of HCC. This study investigated the association between a SNP in PRKCI and the Pakistani population's hepatocellular carcinoma (HCC) risk. Obtained samples were first evaluated for ALT measurements and viral load quantification through reverse transcriptase-PCR. The PKCι nsSNP rs1199520604 was evaluated computationally by multiple consensus bioinformatics tools for predicting its potential deleterious effects. Its association with hepatitis C virus- (HCV) mediated HCC was then investigated through ARMS-PCR (Amplification Refractory Mutation System Polymerase Chain Reaction). SNP analysis of rs1199520604 was performed in 100 cases and 100 controls. Variant rs1199520604’s homozygous T genotype is a risk factor allele for the HCV-induced HCC (odds ratio: 4.13, relative risk: 2.01, *P*-value < 0.0001). The heterozygous genotype is determined to protect HCV patients from HCC development (*P* < 0.001). The study highlighted the disease association of variant rs1199520604 with HCV-induced HCC in the Pakistani populations. This variant, after further validation through high-throughput investigation on a larger cohort, has the potential to be translated at the clinical level.

## Introduction

Among cancer-related deaths, liver cancer is the second leading cause of worldwide deaths. Hepatocellular carcinoma (HCC) is the most frequently occurring type of liver cancer, especially in developing countries^1^ due to its complexity in diagnosis, chemo-resistance, and aggressive nature. It begins in hepatocytes and accounts for 75–85% of all liver cancer cases. Its occurrence rates change from 5.1 per 100,000 person-years in Europe to 17.7 per 100,000 in eastern Asia^2^. Pakistan alone accounted for 4354 new cases and 4365 deaths due to liver cancer in 2020. These disparate rates between regions highlight differences in the occurrence of risk factors^3^. In addition to other risk factors, one of the prominent factors associated with HCC development is Hepatitis C Virus (HCV)^4^.

Single nucleotide polymorphism (SNP) is a common genetic variation among individuals. SNPs have arisen as genetic markers for diseases, and numerous SNP markers are available in public databases. Previous studies have shown the importance of defining mutations as deleterious or non-deleterious and their association with certain diseases. SNPs in the genes GRIK1, MICA, HLA-DQA/DQB, and KIF1B have been reported to be associated with the risk of HCC^5^. The PKC family proteins have been positively associated with HCC in several studies^6–8^. However, those studies usually indicated its involvement in the carcinogenic process at a functional level. Any genetic association, such as the presence of SNPs in the PKCι coding gene (PRKCI) and HCC susceptibility and progression, is not yet reported.

PKCι is a lipid-dependent serine/threonine kinase. It participates in several signaling pathways that regulate cell survival^9–11^, differentiation^10^, polarity^12^, and microtubule dynamics in the early secretory pathway^13^. PKCι belongs to the evolutionarily conserved PKC family. It has 596 amino acids, and its molecular mass is 68,262 Da. PKCι activity is regulated by lipid second messengers (ceramide, phosphatidylinositol 3,4,5-triphosphate, and phosphatidic acid), phosphoinositide-dependent kinase (PDK1), tyrosine phosphorylation, and specific protein–protein interactions. Compared to other isozymes of PKC, PRKCI is more conserved from an evolutionary point of view. The overall amino acid sequence homology of PKCι and PKCζ is 72%, while the kinase domains are 86% identical. PKCι shows less homology with the other isoforms of PKC; for example, even in the highly conserved catalytic domain, it is only 53% identical with other PKC family proteins^14^. Polymorphisms in the PKC gene family have been previously associated with the occurrence of thyroid follicular neoplasms and fibrosarcoma. Non-synonymous SNPs in the PKC family have also been linked with fibroblast transformation. PRKCI genetic associations, such as the presence of SNPs in the coding gene and HCC susceptibility and progression, have yet to be reported. Considering the unique oncogenic potential of PRKCI and the gap in data regarding the role of its variants in HCC progression, we decided to investigate the association between a high-risk PRKCI variant rs1199520604 and HCV-induced HCC in the current study.

## Materials and methods

### SNP selection

The variants data was retrieved from ENSEMBL (ENSEMBL gene ID: ENSG00000163558). Missense variants were sorted and mapped on transcript ENST00000295797. Using ENSEMBL obtained data from different consensus tools (SIFT, PolyPhen2.0, MutationAssssor, CADD, REVEL, and MetaLR), the pathogenicity of the variants was predicted. The criteria for variant classification into disease-causing or tolerant class was chosen from the^15^ study. The variant predicted to be the most pathogenic was selected for further validation.

### Primer designing

ARMS PCR (Amplification Refractory Mutation System Polymerase Chain Reaction) primers against the identified potential common damaging SNP rs1199520604 were designed using primer 1 software^16,17^. The chromosome assembly used as input in Primer1 was 38.p13.

### Study population

**Figure 1:**
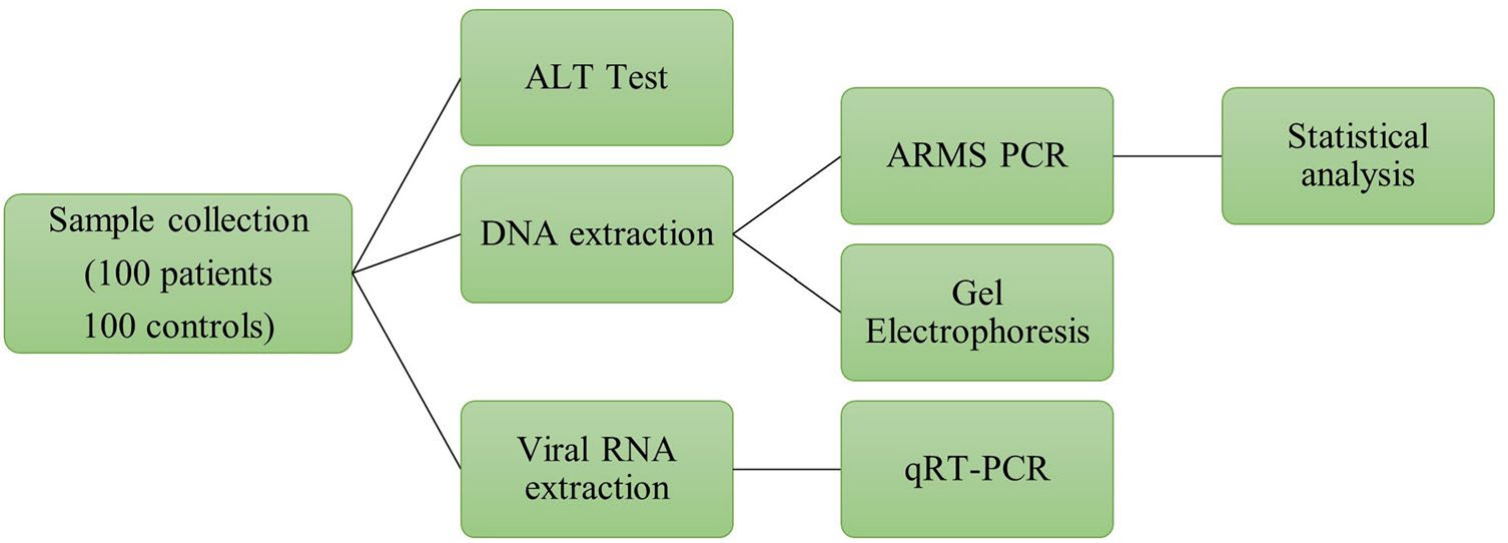
Schematic diagram of different in-vitro methods employed in this study. Samples were collected and processed for the ALT level test. Viral RNA was extracted from HCC patient samples, and qRT-PCR was performed on the extracted RNA. Lastly, genomic DNA was extracted from the samples. The extracted DNA was detected through gel-electrophoresis followed by performing ARMS-PCR and applying statistics on the results of ARMS-PCR.

**Figure 2:**
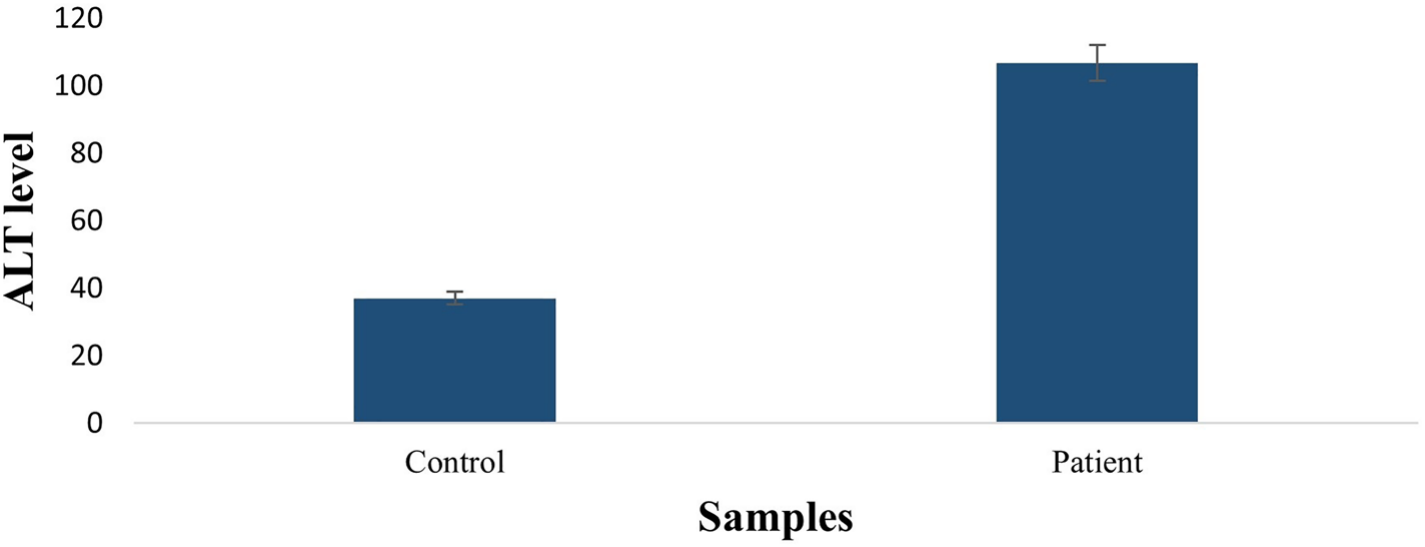
Comparison of ALT concentration in HCC patients and control. ALT level is higher in HCC patients than control samples.

One hundred samples from the HCC patients and 100 control samples were collected for SNP analysis. The ethical review board of ASAB's parent institution, the National University of Sciences and Technology, approved the current study. Blood samples were taken at the Combined Military Hospital in Rawalpindi with verbal and written permission. The research was conducted per the Helsinki declaration's standards and principles. Informed consent was obtained from all subjects and/or their legal guardian(s). Patients with co-morbidity with cardiac or metabolic diseases were excluded, and the sample was collected only from patients having HCV infection (Fig. 1). ALT levels were measured to assess liver function. Patients had significantly higher ALT levels than healthy controls (Result is provided in Fig. 2).

### Genomic DNA extraction and SNP analysis

The method used for Genomic DNA extraction was the phenol–chloroform method^8,18^. ARMS-PCR was performed to detect single nucleotide change in PRKCI. For this type of PCR, specific tetra primers, two internal primers (forward and reverse) and two external primers (forward and reverse) against the selected SNP were used. The SNP-specific primers were inner primers that were used for detection. The sequences for primers are: Forward inner 5′-GTGAAAGCCTACTACCACG-3′, Reverse inner 5′-TCCCAGGACACTCATCA-3′, Outer forward 5′-AGGTGGGCAGGTAGGT-3′, and Outer reverse 5′-CACCCCTATCACTTCGTC-3′. PCR products were run on 2% gel, and band size was determined by comparing it with Thermo Scientific GeneRuler 100 bp DNA Ladder.

### Viral RNA extraction

For the analysis of the viral load in the HCC patients, viral RNA was extracted from the patient’s blood via FAVOREGEN KIT®. For this purpose, 200 µl of serum was combined in a 1.5 mL microcentrifuge tube with 500 µl of VNE buffer. This mixture was vortexed for 5–7 s. 500 µl of 75% ethanol was added and vortexed again for 5–7 s. It was then poured into a spin column and centrifuged at 8000 rpm for 1 min. The spin column was then removed, and the collection tube was discarded. RNA was retained on the filter of the spin column, which was then placed in a new collection tube, and 500 µl of wash buffer 1 was added into the spin column and centrifuged at 8000 rpm for 1 min. The collecting tube was changed, and 750 µl of wash buffer 2 was added and centrifuged at 14,000 rpm for 1 min. The centrifugation step was repeated twice to dry the filter. Lastly, the filter was placed in a new 1.5 mL microcentrifuge tube, and 50 µl of RNase-free water was added. The Eppendorf with the filter was centrifuged at 8000 rpm for 1 min. The RNA eluate was stored at −20 °C.

### Quantitative reverse transcriptase polymerase chain reaction (qRT-PCR) to determine viral load

Blood viral RNA was extracted using the FavorPrepTM Viral DNA/RNA Kit to evaluate the viral load in a patient sample (catalog no. FAVNK 001–1). 200 µl of Blood and 500 µl of VEN buffer were combined in an Eppendorf tube for 5–7 s vortexing. The tube was vortexed for at least 5–7 s after adding 500 µl of 75% ethanol. The material was transported to the spin column and spun for one minute at 8000 rpm. The collecting tube was discarded, and a fresh tube was inserted with the filter tube. After 500 µl of wash buffer 1 was added and centrifuged, the mixture was centrifuged for 1 min at 8000 rpm. The collecting tube was replaced, 750 µl of wash buffer 2 was added, and the tube was centrifuged for one minute at 14,000 rpm. This approach was used twice to dry the filter. Transferred filter tube to Eppendorf tube, added 50 µl of RNase-free water and centrifuged at 8000 rpm for another minute. After transferring the RNA sample to a tube, it was stored at −20 °C.

### Statistical analysis

The collected genotyping data were analyzed statistically using GraphPad Prism 9. The Chi-square test was performed on both the patient and control groups. Furthermore, risk and odds ratios and calculated confidence intervals were measured through Fisher Exact test. Statistical significance was assumed when the *p*-value was less than 0.005.

### Ethics approval

Approval for the study was obtained from the Institutional Review Board of National University of Science and Technology (NUST), Pakistan (IRB No. 10-2021-01/01**).** Informed consent was obtained from all subjects and/or their legal guardian(s).

## Results

### Association of PKCι SNP (rs1199520604) with HCV-mediated HCC

Multiple consensus tools revealed variant rs1199520604 pathogenicity; therefore, this variant was further validated for its association with HCV-mediated HCC. Association was studied in 100 patients and 100 healthy individuals through genotyping PCR. The analysis revealed a significant association of the SNP in homozygous mutated form (i.e., TT) with HCC compared to the heterozygous GT genotype and homozygous wild genotype GG (Table 1; odds ratio 1.134, relative risk 2.012, *P*-value < 0.0001). This shows that the polymorphism with the TT genotype increased the risk of disease occurrence.

**Table 1:** Comparison of PRKCI polymorphism in HCC patients and control.

Genotype	Frequency distribution		Odds ratio		Relative risk		*P* value
	Patients %	Control %	Value	95% CI	Value	95% CI	
GG	23.00	36.00	0.5310	0.2834 to 1.000	0.7138	0.4912 to 0.9914	0.0623
TT	65.00	31.00	4.134	2.247 to 7.278	2.012	1.501 to 2.750	< 0.0001
GT	12.00	33.00	0.2769	0.1349 to 0.5600	0.4697	0.2767 to 0.7418	0.0006

### Gender based association of PKCι SNP (rs1199520604) with HCV-mediated HCC

A comparison of PRKCI polymorphism with HCC patients and controls was performed (Table 2). The data obtained support the results described in Table 1. In both males and females, the mutated homozygous allele TT was found to be associated with the disease. The *P*-value for males and females having allele TT was 0.0006 and 0.0015, respectively, emphasizing the significance of the results.

**Table 2:** Comparison of PRKCI polymorphism in HCC patients and control with respect to gender.

Genotype	Frequency distribution		Odds ratio		Relative risk		*P* value
	Patients %	Control %	Value	95% CI	Value	95% CI	
GG (F)	26.00	40.74	0.5111	0.2130 to 1.223	0.6927	0.4145 to 1.082	0.1466
TT (F)	64.00	31.48	3.869	1.701 to 8.831	1.995	1.319 to 3.116	0.0015
GT (F)	10.00	27.78	0.2889	0.1088 to 0.8573	0.4667	0.2046 to 0.9125	0.0260
GG (M)	20.00	30.43	0.5714	0.2282 to 1.398	0.7500	0.4262 to 1.182	0.2505
TT (M)	66.00	30.43	4.437	1.802 to 10.01	2.024	1.350 to 3.164	0.0006
GT (M)	14.00	39.13	0.2532	0.1011 to 0.6524	0.4623	0.2307 to 0.8207	0.0058

### Relationship of PKCι SNP rs1199520604 alleles with viral load in HCV induced HCC patients

**Figure 3:**
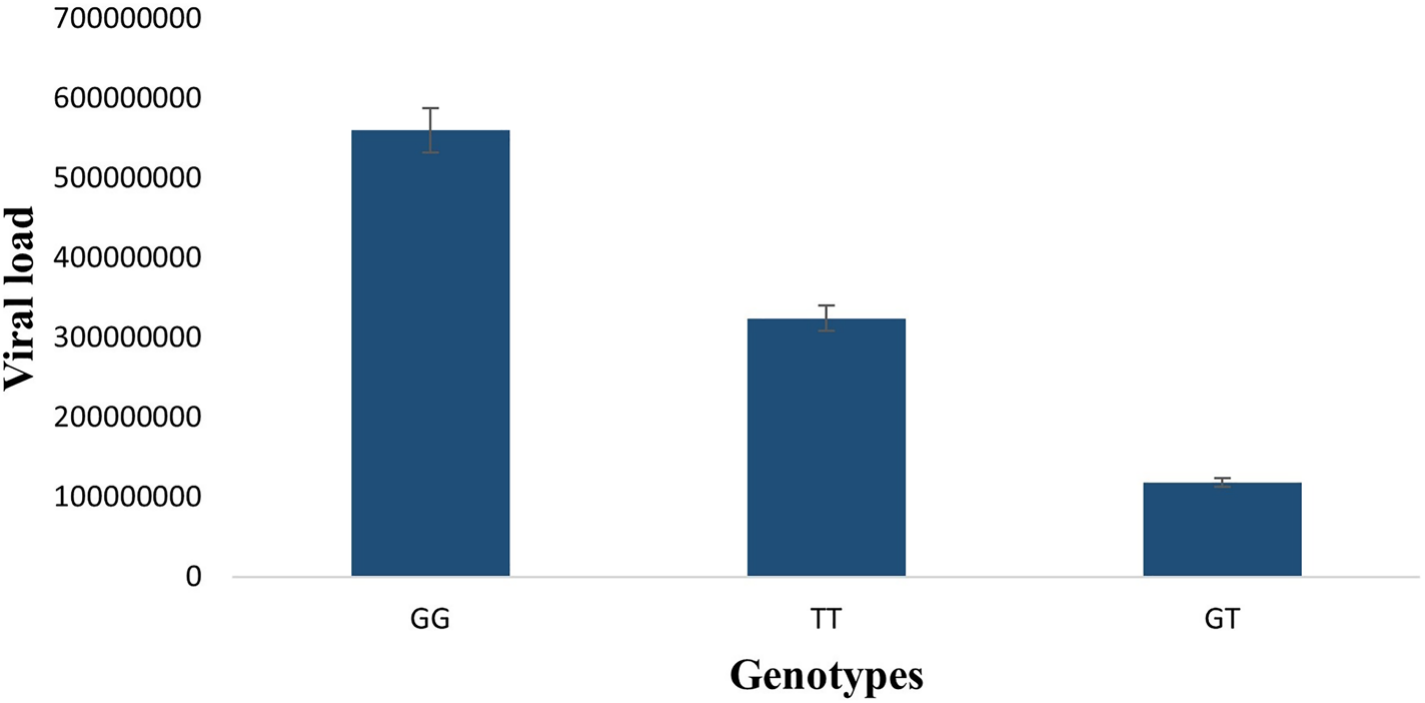
HCV Viral load plotted against homozygous wild (GG), heterozygous (GT) and homozygous mutated (TT) genotypes of HCC patients. GG allele has significantly high viral load compared to TT and GT alleles.

Viral titer in patients was measured by qRT-PCR. The analysis of viral load against PKCι SNP rs1199520604 alleles shows significant differences. Average viral copy number for alleles GG, TT, and GT of rs1199520604 were determined and then analyzed, which shows that patients with GG allele have higher (560,095,306.7 copies/ml) viral copy number compared to patients with TT and GT alleles, which suggests that there may be a correlation of viral load and rs1199520604 genotypes (Fig. 3).

## Discussion

Several genetic and environmental factors are involved in causing hepatocellular carcinoma (HCC). In addition to other risk factors, one of the prominent factors associated with HCC progression is the hepatitis C virus (HCV)^4^. HCV-induced HCC occurrence is increasing globally, particularly in developing countries. A significant problem with most cancers is detecting the disease in its early stages, as is the case for HCC. The change in protein expression and its structural and functional properties have often been associated with the presence of certain kinds of polymorphisms in the genetic sequence^19–21^; however, not much is known about the role of PRKCI variations about natural polymorphism. It is, therefore, crucial to identify deleterious nsSNPs in the PRKCI gene, as nsSNPs cause the most significant damaging impact on the protein structure and function^22^.

The current study only considered missense variants because of their direct involvement in disease pathologies and their impacts on the adopted treatment regimen. So, in this study, SNP in PRKCI was correlated with HCC to identify a new prognostic marker for HCC. The selected SNP (rs1199520604), when processed through different computational tools, was the most deleterious. This SNP (rs1199520604) falls within the PB1 domain (which is exclusive to atypical PKCs) of PKCι^23^. The SNP changes the amino acid Glycine to Tryptophan at position 34. The PB1 domain facilitates protein–protein interactions between PKCι and other proteins having PB1 domain, such as Par-6 (partitioning-defective 6)^12,24^, ZIP/p62^25^, and MEK5 (MAPK (mitogen-activated protein kinase)/ERK (extracellular-signal-regulated kinase) kinase 5)^26^. The PB1 domain is near a highly conserved region, making it a striking therapeutic target for cancer treatment^23^. The analysis of PCR results after amplifying the SNP in 200 samples (100 controls and 100 HCV-induced HCC patients) revealed that the homozygous mutant allele TT had a significant correlation with HCC compared to homozygous GG and heterozygous GT alleles. The analysis of PCR data concerning gender also indicated the significant association of the same alleles in both males and females with the disease. However, the relative risk and odds ratio in males was slightly more significant than in females. This difference could also be notable in the higher incidence rate of HCC in males than in females^27,28^. The association of polymorphism with genetic disease gives us an idea about susceptibility and can also be used for early diagnosis^29,30^. Thus, this SNP (rs1199520604) could be a potential biomarker for the prognosis of HCC.

It has been established that high alanine aminotransferase level is linked with HCV-induced HCC and can lead to rapid disease development^31^. The ALT levels in HCC patients compared to control revealed significantly increased ALT levels in HCC patients (106 IU/L). The association of viral load with rs1199520604 alleles was performed to analyze the link of genotype with viral load. Our results demonstrated that patients with genotype GG have a high viral load, followed by TT and GT. It might be due to clearance of viral load over time as literature shows clearance of HCV viral RNA in patients coinfected with HCV/HIV-1 having rs12979860 polymorphism CC genotype^32^.

## Conclusion

In conclusion, our study demonstrated that the pathogenic SNP (rs1199520604) of PKCι, identified through computational tools, is strongly associated with HCV-induced HCC. This association indicates that the SNP (rs1199520604) may serve as a prognostic marker for HCV-induced HCC. High ALT levels were observed in HCC patients, and a correlation between PKCι SNP rs1199520604 genotypes and viral loads gave insight that there may be an association between them. The expression profile of PKCι upon G34W mutation needs to be further explored to elucidate its role as a prognostic or diagnostic marker and open new therapeutic avenues for HCC treatment. The association between the viral load and PKCι SNP rs1199520604 genotypes need to be studied at the molecular level to explain the correlation better. Further in vitro and in vivo studies are required to ascertain the effects of the variant on native protein structure and function and how tumor progression is affected.

## Data Availability

All the relevant data has been provided in the manuscript used and/or analyzed during the current study are available from the corresponding author on reasonable request.
